# New-onset diabetic ketoacidosis in a 13-months old african toddler: a case report

**DOI:** 10.11604/pamj.2015.22.293.7581

**Published:** 2015-11-24

**Authors:** Jean-Claude Katte, Romance Djoumessi, Gisele Njindam, Gerard Tama Fetse, Mesmin Dehayem, Andre-Pascal Kengne

**Affiliations:** 1 Bafoussam Regional Hospital, Ministry of Public Health, Bafoussam, Cameroon; 2 Yaoundé Central Hospital, Diabetes and Metabolic Disease Unit, Cameroon; 3 Department of Medicine, Faculty of Health Sciences, University of Cape Town, Cape Town, South Africa; 4 Non-communicable Disease Research Unit, South African Medical Research Council& University of Cape Town, PO Box 19070, Tygerberg, Cape Town 7505, South Africa

**Keywords:** Diabetic ketoacidosis, children, Africa, diabetes complication

## Abstract

Type 1 diabetes mellitus is very rare in infants and toddlers and is usually associated with high mortality when complicated with diabetic ketoacidosis (DKA). Toddlers in DKA are often missed in our typical African setting where there is low index of suspicion. Usually, the classical symptoms are not usually at the forefront and many infants and toddlers who develop DKA are mistreated for infections. The case of a 13-months old toddler with new-onset type 1 diabetes mellitus, complicated with DKA at diagnosis is reported in view of its rarity and elevated mortality even when diagnosed in our African setting. She was subsequently treated with intravenous insulin and was passed over to subcutaneous insulin after the eradication of ketones in urine. She continues follow-up at the out-patient children diabetes clinic at the Bafoussam Regional Hospital.

## Introduction

Toddlers constitute a minority of individuals with type 1 diabetes. There exist evidence from epidemiological data suggesting a trend towards type 1 diabetes diagnosis at a younger age [1]. These very young children pose significant challenges to both the health care providers and families. The family of the infant or toddler with type 1 diabetes is faced with a long and seemingly arduous journey through early childhood, the school years and adolescence before adulthood when the patient is less reliant on the family members for the routine management of their condition [2]. At diagnosis, usually the classical symptoms of polyuria and nocturia, polydipsia, polyphagia, and weight loss are often overlooked or ascribed to other causes until the disorder has progressed to overt diabetic ketoacidosis (DKA) [3]. Toddlers in DKA may be misdiagnosed as having pneumonia, asthma, or bronchiolitis and therefore treated with glucocorticoids which could exacerbate the metabolic disorder. Since the diagnosis in most cases is not suspected as it evolves, the duration of symptoms may be longer, leading to more severe dehydration and acidosis and ultimately to coma [4]. DKA is not uncommon at diagnosis in children and infant with diabetes. Even in developed countries, some 15-70% of all newly diagnosed infants and children with diabetes present with DKA [3]. The frequency of DKA in newly diagnosed diabetes globally varies regionally from 11% to 67% in Europe to 28.4% in the USA [5]. The true incidence of DKA in sub-Saharan Africa is unknown but has been estimated at 24% by the International Diabetes Federation (IDF) in 2011, suggesting that many cases are underreported or misdiagnosed leading to a very high mortality in this setting [6]. Here we report the case of a toddler diagnosed with new-onset diabetic ketoacidosis at the emergency department of the Bafoussam Regional Hospital in Cameroon.

## Patient and observation

NP, a 13 months toddler was admitted to the Emergency Department of the Bafoussam Regional Hospital, with the complaints of lack of energy, fever, and cough for 7 days. She has never been sick or admitted before her recent febrile crisis. She feeds of breast milk and other household meals. There is no family history of diabetes mellitus.

On examination, the patient is ill-looking, with deep and slow respiration, temperature of 38.5^°^C, pulse rate of 122 per minute, and weight of 10kg. She presented with an altered mental status, with mild anemia and icterus. On abdominal examination liver was enlarged at 3cm below the costal margin and spleen was not palpable. Investigations showed: Random blood glucose 556mg/dL, total leukocyte count 13 000/µl (differential count - neutrophils: 80%, lymphocytes: 10%, eosinophils: 6%, monocytes: 1%, basophils: 1%) hemoglobin 9.9g/dL, platelets 112 00/µl, C-reactive protein 24mg/L, blood urea 20.0 mg/dL, serum creatinine 1.1 mg/dL, aspartate-amino transferase 88UI/L, alanine-amino transferase 41UI/L, amylase 59UI/l, glycated hemoglobin (A1c) 13.2%, lipid profile: total cholesterol 1.20g/L, triglycerides 0.63g/L, LDL 0.84g/L, HDL 0.25g/L, calcemia 85mg/L, magnesemia 19mg/L, kalemia 2.2mmol/L, natremia 141mmol/L, chloremia 108mmol/L. Urine examination showed the presence of reducing sugar, protein, ketone bodies, no leukocyte, no nitrites. No germ was grown in culture.

The diagnosis of newly-onset type 1 diabetes mellitus complicated with ketoacidosis was made. She was treated with isotonic saline 0.9%, and human insulin with other supportive measures. Human insulin was administered intravenously and continuously using an electric syringe. The blood glucose level was normalized with IU of insulin received within 48 hours, and followed by a complete eradication of ketone bodies in the urine. She was maintained on the intravenous insulin perfusion using the electric syringe with isotonic saline and Dextrose to prevent hypoglycemia. She was later started out on pre-mixed insulin (10 IU) twice per day. She recovered and was discharged on the 11th day. She continued insulin at home but presented with multiple episodes of hypoglycemia 5 months later and insulin was stopped. The HbA1c was 7.8%. She stayed for 3 months without insulin but later presented with hyperglycemia and pre-mixed insulin was reintroduced at 4 IU per day. HbA1c performed 3 months after insulin after the reintroduction of insulin was 8.9% and the insulin was titrated to 5 IU per day in 2 months.

## Discussion

DKA is defined by blood glucose concentrations >200 mg/dL, venous blood pH <7.3, bicarbonate <15 mM, glycosuria, ketonemia and ketonuria. DKA is a consequence of absolute or relative lack of insulin with a concomitant rise in plasma levels of insulin counter-regulatory hormones such as catecholamines, glucagon, cortisol and growth hormone [7]. DKA is the most common hyperglycemic emergency in patients with type 1 diabetes mellitus. This acute diabetic complication has a high death-to-case ratio of 5 to 10% [8]. In Africa, the mortality of DKA is unacceptably high with reported death rate of 26 to 29% in studies from Kenya, Tanzania and Ghana [6].

DKA in this toddler was diagnosed based on the hyperglycemia greater than 400 mg/dL, ketones - glucose in urine and her clinical presentation. She probably had a concomitant upper respiratory tract viral infection since she had an intermittent fever and cough that started seven days before her presentation at the emergency department. Research findings have shown that the most common precipitating factor DKA occurrence is infection [9]. The total leucocyte count was slightly raised and with an elevated CRP at 24 mg/L confirming the probable presence of an inflammatory process. No parasitic or bacterial infection was isolated. It is worth noting that, other precipitating factors are the omission of insulin and undiagnosed diabetes. However, some compounding precipitating and risk factors for DKA specific to our African settings apart from infections are poor healthcare delivery systems, lack of education and malnutrition [10]. She was immediately treated with insulin and fluid replacement therapy which according to literature, remain the mainstay of treatment for children with DKA although it has not been standardized and remains controversial. Contentious issues include the amount and type of intravenous fluids to be used and rate of delivery although normal saline is usually preferred considering the low cost. We used a low dose (0.1 IU/kg/hour) continuous IV administration of soluble insulin scheme in order to reduce the chances of a severe hypoglycemia which will be deleterious for the brain of the toddler. Low-dose insulin IV administration is currently the standard of care in patients with DKA so as to reduce the possibility of the development of cerebral edema which accounts for the majority of all DKA-related deaths in children.

After the resolution of DKA, she was continued on insulin and dietary care, with daily education sessions with her mother in order to help the latter better manage the toddler at home. At this early age where the treatment at home of the toddler totally depends on her family, it is therefore important to arm the parent or carer with the necessary information as concerns feeding, insulin treatment and most especially the recognition and the management of hypoglycemia.

## Conclusion

Despite recent advances in medical sciences, DKA remains a major concern in sub-Saharan Africa. Poverty, poor healthcare delivery system, ignorance and infectious disease burden impede the management of DKA. Most importantly, type 1 diabetic patients and their care givers should be trained on the importance of blood glucose and urinary ketone monitoring and to educate the general population as a whole.

## References

[1] Gardner SG, Bingley PJ, Sawtell PA, Weeks S, Gale EA (1997). Rising incidence of insulin dependent diabetes in children aged under 5 years in the Oxford region: time trend analysis: The Bart's-Oxford Study Group. Bmj..

[2] Daneman D, Frank M, Perlman K, Wittenberg J (1999). The infant and toddler with diabetes: Challenges of diagnosis and management. Paediatrics & Child Health..

[3] Wolfsdorf J, Glaser N, Sperling MA (2006). Diabetic ketoacidosis in infants, children, and adolescents: a consensus statement from the American Diabetes Association. Diabetes Care..

[4] Young E, Bradley RF (1967). Cerebral edema with irreversible coma in severe diabetic ketoacidosis. N Engl J Med..

[5] Rosenbloom AL (2010). The management of diabetic ketoacidosis in children. Diabetes Ther..

[6] Otieno CF, Kayima JK, Omonge EO, Oyoo GO (2005). Diabetic ketoacidosis: risk factors, mechanisms and management strategies in sub-Saharan Africa: a review. East Afr Med J..

[7] American Diabetes Association (2001). Position statement: hyperglycemic crises in patients with diabetes mellitus. Diabetes Care 24.

[8] Faich GA, Fishbein HA, Ellis SE (1983). The epidemiology of diabetic acidosis: a population-based study. Am J Epidemiol..

[9] Morris AD, Boyle DI, McMahon AD, Greene SA, MacDonald TM, Newton RW (1997). Adherence to insulin treatment, glycaemic control, and ketoacidosis in insulin-dependent diabetes mellitus, The DARTS/MEMO Collaboration, Diabetes Audit and Research in Tayside Scotland, Medicines Monitoring Unit. Lancet..

[10] Van Zyl DG (2008). Diagnosis and treatment of diabetic ketoacidosis. SA Fam Pract..

